# Data on the characterization of follicle-stimulating hormone monoclonal antibodies and localization in Japanese eel pituitary

**DOI:** 10.1016/j.dib.2016.05.069

**Published:** 2016-06-03

**Authors:** Dae-Jung Kim, Chae-Won Park, Munkhzaya Byambaragchaa, Shin-Kwon Kim, Bae-Ik Lee, Hyung-Kyu Hwang, Jeong-In Myeong, Sun-Mee Hong, Myung-Hwa Kang, Kwan-Sik Min

**Affiliations:** a Aquaculture Research Division, National Institute of Fisheries Science (NIFS), Busan 46083, Republic of Korea; b Animal Biotechnology, Graduate School of Future Convergence Technology, Institute of Genetic Engineering, Hankyong National University, Anseong 17579, Republic of Korea; c Dept. of Research and Development, Institute of Gyeongbuk Marine Bioindustry, Ulgin, 36315, Republic of Korea; d Department of Food Science & Nutrition, Hoseo University, Asan 31499, Republic of Korea

**Keywords:** Japanese eel, FSH, Monoclonal Antibody

## Abstract

Monoclonal antibodies were generated against recombinant follicle-stimulating hormone (rec-FSH) from Japanese eel *Anguilla japonica*; rec-FSH was produced in *Escherichia coli* and purified using Ni-NTA Sepharose column chromatography.

In support of our recent publication, "Production and characterization of monoclonal antibodies against recombinant tethered follicle-stimulating hormone from Japanese eel *Anguilla japonica*" [1], it was important to characterize the specificity of eel follicle-stimulating hormone antibodies. Here, the production and ELISA system of these monoclonal antibodies are presented. The affinity-purified monoclonal antibodies specifically detected eel rec-FSH in ELISA and on western blots of rec-FSH produced from CHO cells. Immunohistochemical analysis revealed that FSH staining was specifically localized in the eel pituitary.

**Specifications Table**

**Table:** 

Subject area	*Biology*
More specific subject area	*Eel FSH antibody*
Type of data	*Figures, graphs, tables and Western blots*
How data was acquired	*ELISA, Western blotting and immunohistochemistry*
Data format	*Analyzed*
Experimental factors	*Immunization of mice with rec-eel FSH*β*/*α*, antibody purification and isotype determination*
Experimental features	*Characterization of monoclonal antibody and ELISA analysis using purified antibody, western blotting and confocal microscopy to determine the localization of FSH in the pituitary*
Data source location	*Anseong and Busan, Korea*
Data accessibility	*Data presented in this article*

**Value of the data**

• The antibody generated can serve as a tool for basic research in the field of eel FSH biology.
• FSH localization in the pituitary suggests a potential FSH role during oocyte- maturation.
• ELSIA system can analyze the quantity of rec-eel FSH hormone and be used in investigations in reproductive endocrinology *in vitro* and *in vivo*.

## 1 Data

**Fig. 1:**
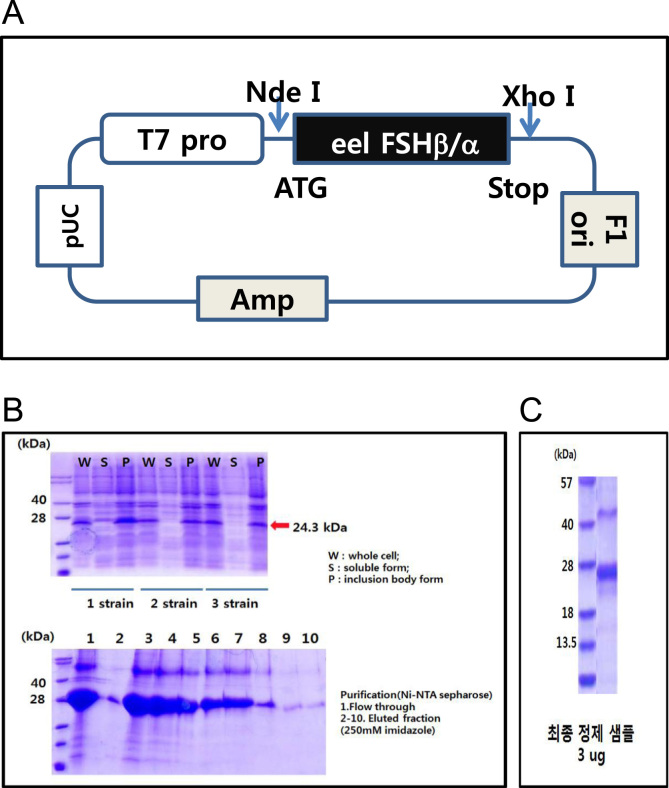
Construction of eel FSHβ/α expression vector and expression of rec-FSHβ/α in *E. coli*. (A) Construction of rec-tethered eel FSHβ/α cDNA by using overlapping PCR. The expression vector was subcloned into pRSET under the T7 promoter (designated as pRSET-eel FSHβ/α). (B) The pRSET vector was transformed into *E. coli*. After culture, whole-cell, soluble, and inclusion-body fractions were subjected to SDS-PAGE. One strain was cultivated in large-volume cultures and the protein was purified using Ni-NTA Sepharose column chromatography.

**Fig. 2:**
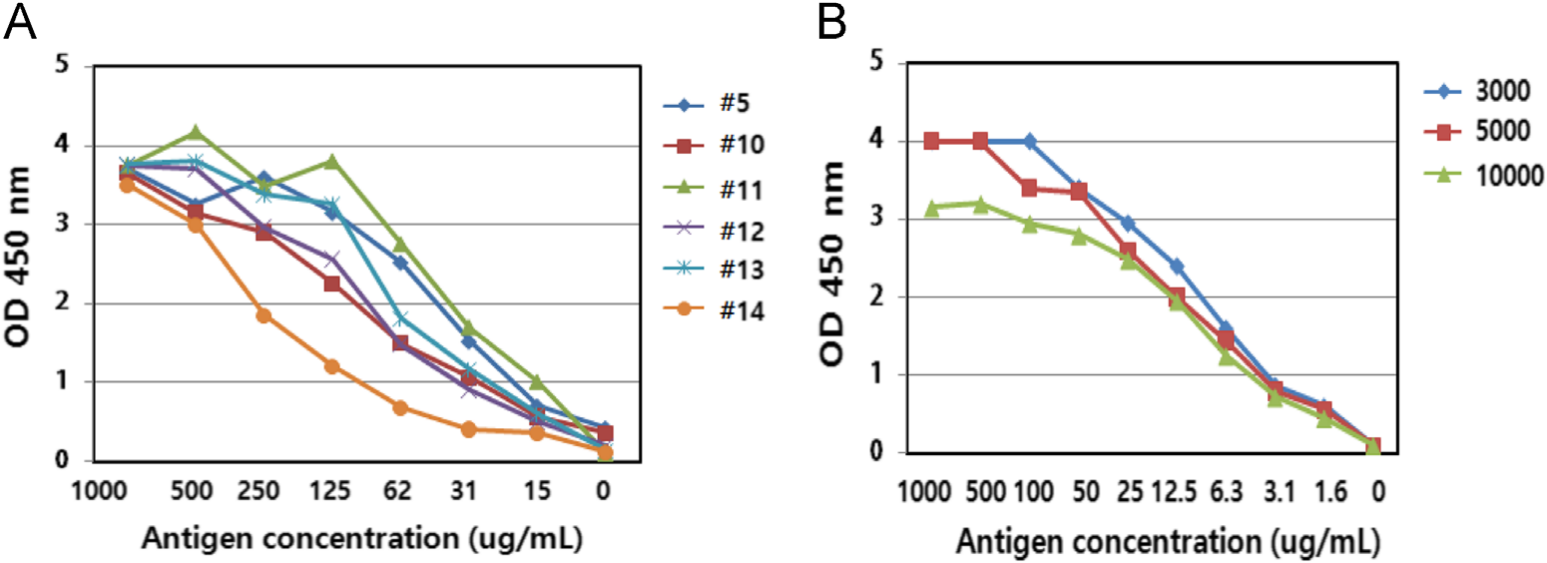
Indirect ELISA for testing the reactivity of antibodies. The reactivity of culture supernatants from selected hybridoma cells was checked by performing indirect ELISA according to the protocol described in Section 2.5. Six positive clones were selected based on testing the supernatants of the hybridoma cells.

**Fig. 3:**
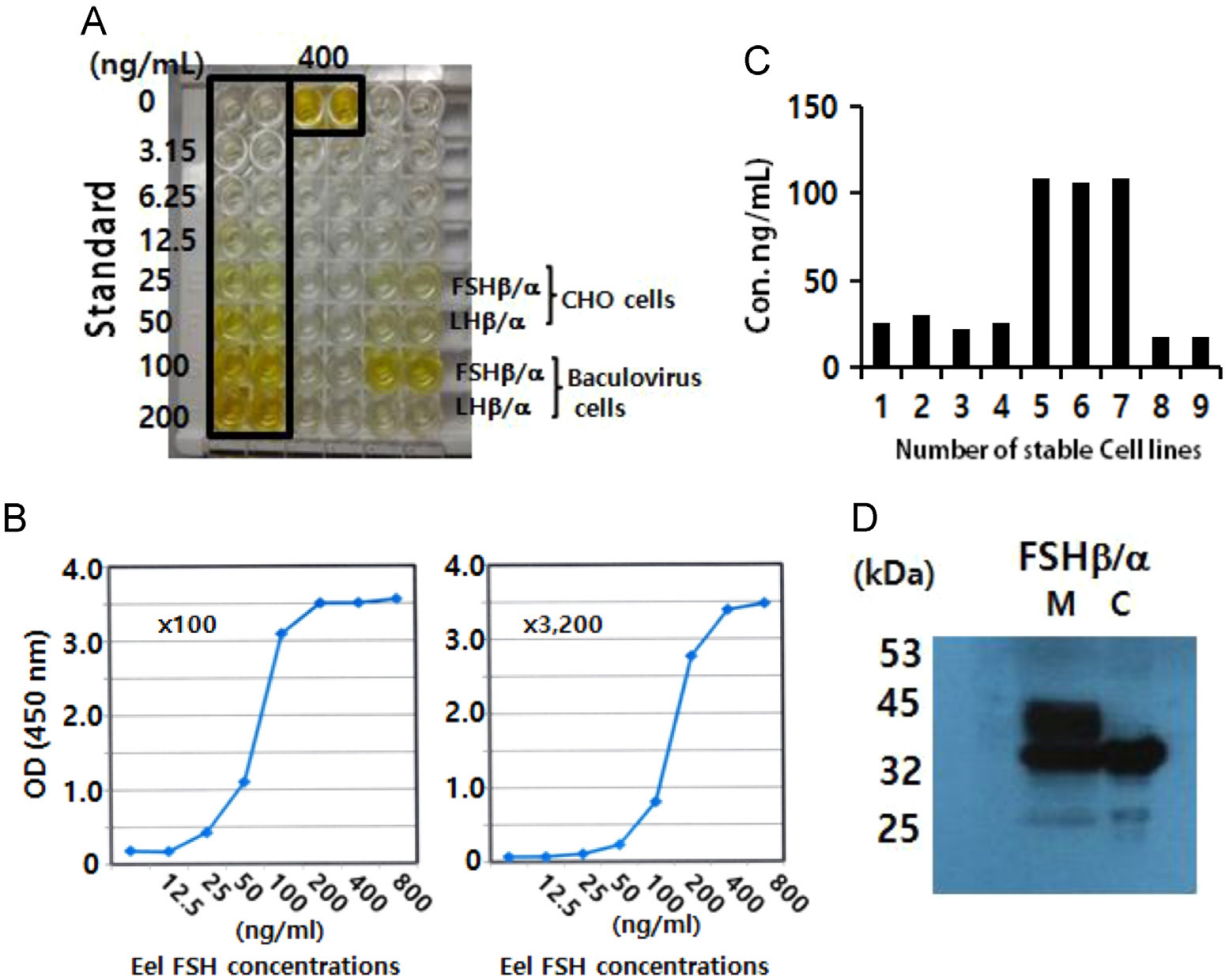
Quantification of rec-FSHβ/α and LHβ/α amounts by using sandwich-ELISA system. (A) Proteins (rec-FSHβ/α and LHβ/α) produced from CHO cells and baculovirus-infected cells were analyzed by performing sandwich ELISA. (B) The HRP-conjugated anti-eel FSH monoclonal antibody eFA-C11, diluted 100–3200 times, was tested; this dilution range of the HRP-labeled antibody efficiently described the standard curve. (C) Quantities of rec-FSHβ/α produced from stable cell lines were also determined. (D) Western blotting performed using eFA-C5 antibody is shown here. The 2nd antibody used was goat anti-mouse (1:3000). The monoclonal antibody detected a single ~34-kDa FSHβ/α band in the medium (M) and cell lysates (C).

**Fig. 4:**
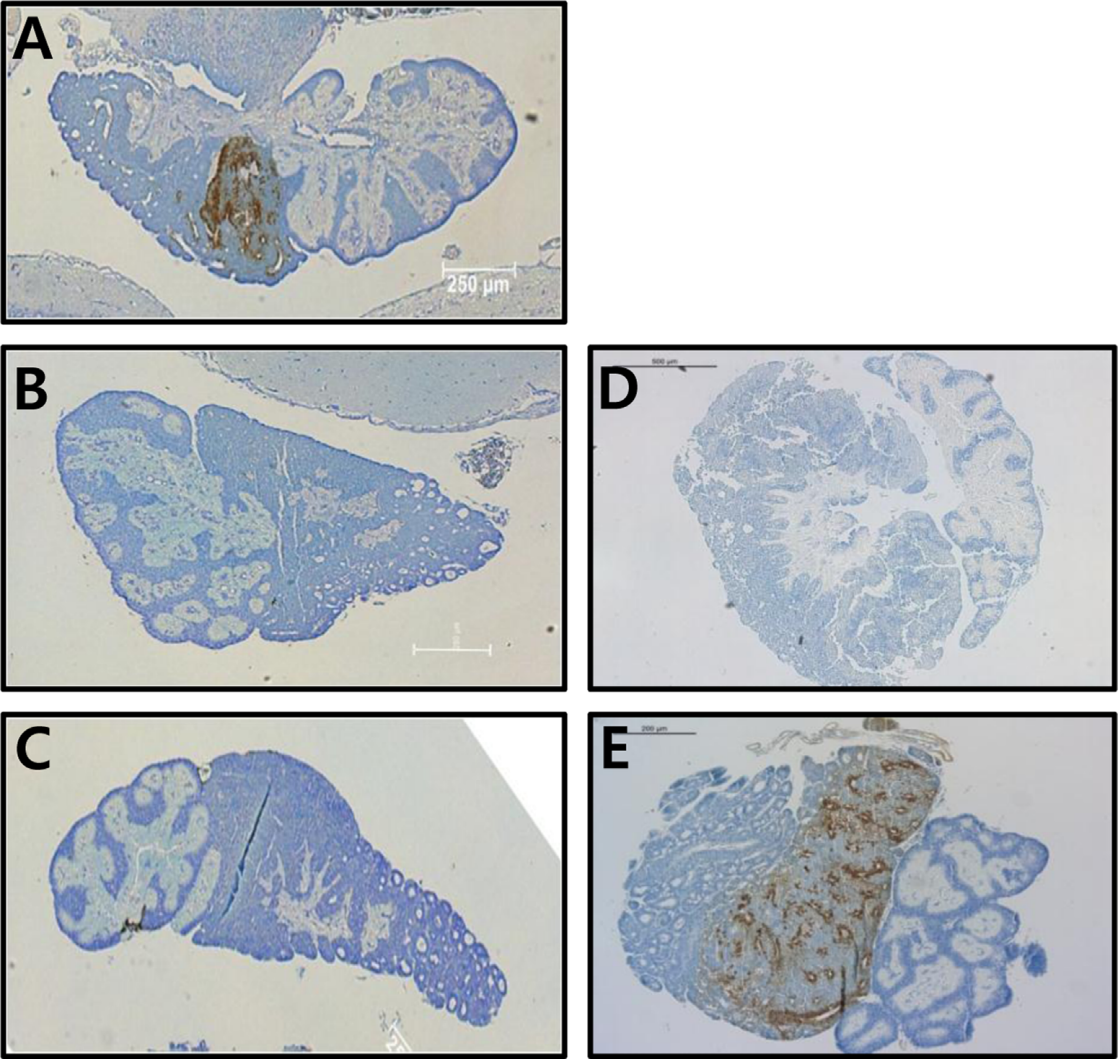
Localization of FSHβ-subunit expression in the eel pituitary during oocyte-maturation. Adjacent sections of the pituitary were stained with eFA-C14, an antiboy that specifically binds to eel FSHβ-subunit, and detection was performed using swine anti-mouse IgG secondary antibodies (1:1000). Representative IHC analyses are shown. The staining revealed the specific site of FSHβ-subunit in the pituitary. (A) GSI>0.5%; (B) 5.7%; (C) 33.8% (immediately before ovulation); (D) immediately after ovulation; (E) on Day 7 after ovulation.

The pRSET expression vector encoding a putative protein containing 220 amino acids was constructed (Fig. 1A). The protein in *Escherichia coli* was purified using a 1^st^ Ni-NTA Sepharose column and a 2nd Sepharose column (Fig. 1B,C). After mice were immunized with the antigen, the supernatants of the hybridoma cells were analyzed by using indirect ELISA (Fig. 2). To establish a sandwich-ELISA system, the intersection method was used with HRP-labeled antibodies (Table 1). The quantities of rec-FSHβ/α and luteinizing hormone (LH) β/α and the selected stable cell lines, and western blot result were described **(**Fig. 3**).** The FSHβ-subunit antibody (eFB-C14) was used for examining FSH localization in the pituitary during oocyte maturation **(**Fig. 4**)**.

## 2 Experimental design, materials and methods

### 2.1 Experimental design

A cDNA encoding eel FSHβ/α was cloned into the vector pRSET, one *E. coli* strain expressing FSHβ/α was selected and cultivated in large-volume cultures. The protein was purified and immunized, and then spleen cells were fused with Sp2/0 cells. Subsequently, hybridoma cells were selected. The reactivity of the culture supernatant was tested using indirect ELISA, and the antibodies were purified using Hi-Trap Protein G columns. The antibodies were tested for specificity by performing sandwich-ELISA analysis, western blotting, and immunohistochemical analysis.

### 2.2 Construction of vector for eel FSHβ/α expression in E. coli and CHO cells

The primers for eel FSHα-subunit and FSHβ-subunit were designed based on their nucleotide sequence [2]. Total RNA was extracted from eel pituitary tissues and the cDNAs encoding the rec-tethered eel proteins were constructed as described previously [3] with modifications. The rec-tethered eel FSHβ/α cDNA was amplified using primers containing *Nde*I/*Xho*I sites and subcloned into the pRSET expression vector (Fig. 1A). To express rec-FSHβ/α protein in CHO cells, the cDNA was ligated into the same sites of pcDNA3.1 expression vector.

### 2.3 Production of eel FSHβ/α in *E. coli*

pRSET vector was transformed into *E. coli*. After culture, whole-cell lysates, the soluble fraction, and the inclusion-body fraction were subjected to SDS-PAGE (Fig. 1B). The protein was detected in whole-cell lysates and the inclusion-body fraction from all strains. Strain 2 showed the highest protein levels in the whole-cell lysates and was thus cultivated in large-volume cultures. The protein was purified by performing Ni-NTA Sepharose column chromatography with 250 mM imidazole (Fig. 1B). The final collected fraction (3 μg) was subjected to SDS-PAGE (Fig. 1C).

### 2.4 Generation of monoclonal antibodies and indirect ELISA

Antibodies were manufactured according to a previously reported method used in our laboratory [1], [4]. Mice were immunized with the purified eel rec-FSHβ/α, and spleen cells from the immunized mouse presenting the highest antibody titer were mixed with Sp2/0 cells and then hybridoma cells were selected. The reactivity of the culture supernatant was examined using indirect ELISA. Finally, the optical density (OD) of the reaction mixtures was measured at 450 nm by using an ELISA reader (BioTek, Winooski, VT, USA). Six positive clones were selected (#5, #10, #11, #12, #13, and #14) **(**Fig. 2**)** and the antibodies (#5, #11, and #14, designated as eFA-C5, eFA-C11, and eFB-C14) were purified using Hi-Trap Protein G columns (data not shown).

### 2.5 Establishment of sandwich-ELISA analysis system

To establish an ELISA analysis system by using our monoclonal antibodies, eFA-C5, eFA-C11, and eFB-C14 were coated in 96-well plates and incubated overnight at 4 °C. The dilution samples of the antigen (5–320-times diluted) were added and incubated for 1 h. HRP-conjugated anti-eel FSH monoclonal antibodies were added by intersection. After washing, OD values were measured at 450 nm as described in the preceding section (Table 1A). Next, the rec-proteins were used to establish the sandwich analysis system. The antibodies eFA-C5 and eFA-C11 were coated, and then 20–1280-fold-diluted samples of rec-FSHβ/α or rec-LHβ/α were added and incubated for 1 h at 37 °C. Thereafter, the procedure was as described above for ELISA analysis. The sandwich-ELISA results showed that the optimal combination of 2 antibodies was eFA-C5 for coating plus HRP-conjugated eFA-C11 for detection (Table 1B).

### 2.6 Analysis of rec-tethered eel FSHβ/α by using sandwich ELISA and Western blotting

Cultured CHO-K1 cells were transfected with the expression vectors by using the liposome transfection method as described previously [3]. As the standard in the assays, the antigen was used at 0–400 ng/mL. The proteins (rec-FSHβ/α and LHβ/α) produced from CHO cells and baculovirus-infected cells were analyzed using sandwich ELISA. The baculovirus samples were obtained from the Institute of Gyeongbuk Marine Bioindustry, Ulgin, Korea (Dr. Hong). The results were shown in Fig. 3A. The HRP-conjugated anti-eel FSH monoclonal antibody eFA-C11, diluted 100 and 3200 times, was tested (Fig. 3B)**.** The quantities of rec-FSHβ/α produced from stable cell lines were also analyzed **(**Fig. 3**C).** Furthermore, cells media and lysates were collected and subjected to western blotting. This antibody detected a single prominent ~34-kDa FSHβ/α band against a clean background on western blots of the media and lysates **(**Fig. 3**D).**

### 2.7 Immunohistochemistry of eel pituitary glands

Immunohistochemical (IHC) staining of eel pituitary samples [gonadosomatic indices (GSI)<0.5%, 5.7%, and 33.8% (immediately before ovulation), immediately after ovulation, and on Day 7 after ovulation] was performed using the Vectastain ABC kit according to the method described previously [4]. The sections were incubated overnight at 4 °C with the primary antibody eFB-C14 (1:500) diluted in 5% horse serum blocking buffer, and then with a biotinylated secondary antibody (polyclonal swine anti-mouse IgG, 1:1000). Tissue sections were immunostained using the ABC detection kit and stained with DAB (Fig. 4)**.**

## Figures and Tables

**Table 1:** Establishment of sandwich-ELISA analysis system. (A) HRP-conjugated anti-eel monoclonal antibodies, diluted 100-fold in PBS, were added by intersection. OD values were measured at 450 nm as described in Section 2. (B) The sandwich-ELISA analysis system was established using rec-FSHβ/α and rec-LHβ/α. The antibodies eFA-C5 and eFA-C11 were coated in 96-well plates and incubated overnight at 4 °C. The standard curve shows that the optimal result was obtained with the combination between eFA-C5 antibody coating (capture) and eFA-C11 HRP labeling (detection).
