# Supplementary figures and images for: Data on the characterization of follicle-stimulating hormone monoclonal antibodies and localization in Japanese eel pituitary
Source: Data Brief. 2016 Jun 3;8:404–10. doi: 10.1016/j.dib.2016.05.069 (PMC4909833; doi:10.1016/j.dib.2016.05.069)

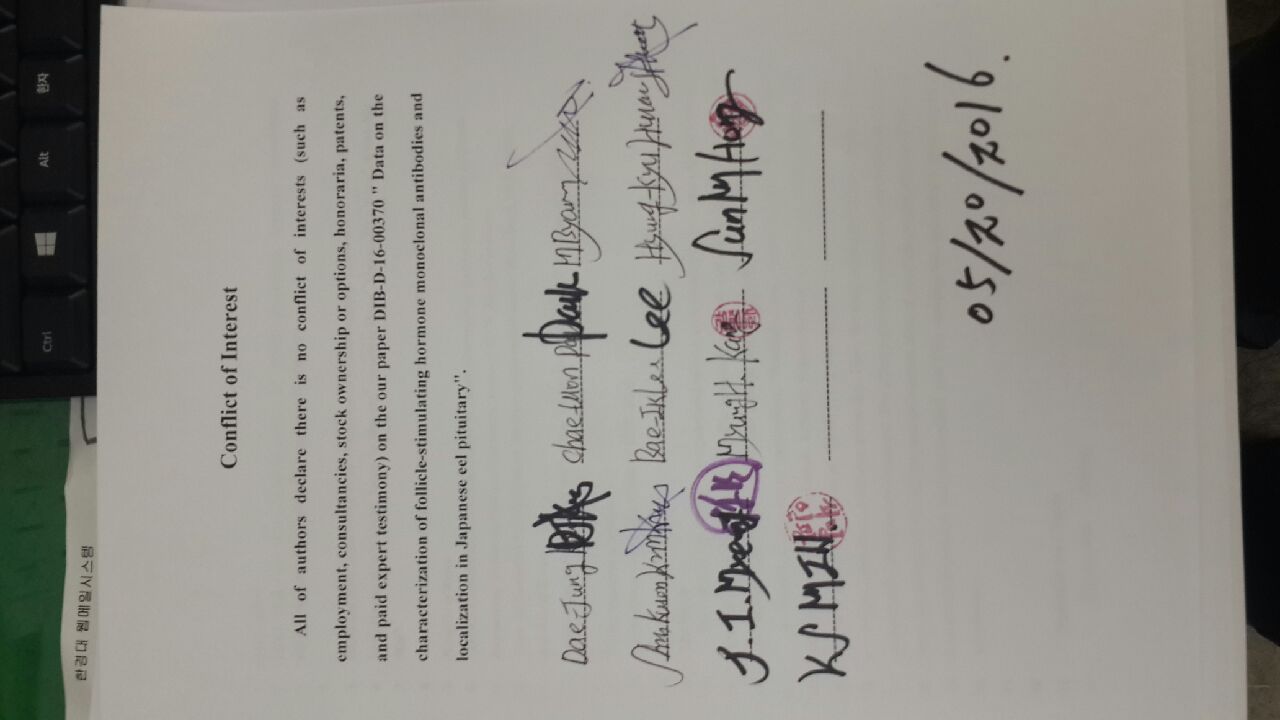

Supplement: Supplementary file 1 — Supplementary material [file mmc1.doc]
